# Capybara Oil Improves Hepatic Mitochondrial Dysfunction, Steatosis, and Inflammation in a Murine Model of Nonalcoholic Fatty Liver Disease 

**DOI:** 10.1155/2018/4956079

**Published:** 2018-04-29

**Authors:** Polyana C. Marinho, Aline B. Vieira, Priscila G. Pereira, Kíssila Rabelo, Bianca T. Ciambarella, Ana L. R. Nascimento, Erika Cortez, Aníbal S. Moura, Fernanda V. Guimarães, Marco A. Martins, Gonzalo Barquero, Rodrigo N. Ferreira, Jorge J. de Carvalho

**Affiliations:** ^1^Laboratory of Ultrastructure and Tissue Biology, Institute of Biology, State University of Rio de Janeiro, Rio de Janeiro, RJ, Brazil; ^2^Biomedical Sciences Department, Ross University School of Veterinary Medicine, Basseterre, Saint Kitts and Nevis; ^3^Laboratory of Stem Cells Research, Institute of Biology, State University of Rio de Janeiro, Rio de Janeiro, RJ, Brazil; ^4^Laboratory of Nutrition and Development Physiology, Institute of Biology, State University of Rio de Janeiro, Rio de Janeiro, RJ, Brazil; ^5^Laboratory of Inflammation, Oswaldo Cruz Institute, Fiocruz, Rio de Janeiro, RJ, Brazil; ^6^Tropical Sustainability Institute, São Paulo, SP, Brazil; ^7^College of Valença, Dom André Arcoverde Educational Foundation, Valença, RJ, Brazil

## Abstract

Nonalcoholic fatty liver disease (NAFLD) is recognized as the most common cause of liver dysfunction worldwide and is commonly associated with obesity. Evidences suggest that NAFLD might be a mitochondrial disease, which contributes to the hepatic steatosis, oxidative stress, cytokine release, and cell death. Capybara oil (CO) is a rich source of polyunsaturated fatty acids (PUFA), which is known to improve inflammation and oxidative stress. In order to determine the effects of CO on NAFLD, C57Bl/6 mice were divided into 3 groups and fed a high-fat diet (HFD) (NAFLD group and NAFLD + CO group) or a control diet (CG group) during 16 weeks. The CO (1.5 g/kg/daily) was administered by gavage during the last 4 weeks of the diet protocol. We evaluated plasma liver enzymes, hepatic steatosis, and cytokine expression in liver as well as hepatocyte ultrastructural morphology and mitochondrial function. CO treatment suppressed hepatic steatosis, attenuated inflammatory response, and decreased plasma alanine aminotransferase (ALT) in mice with NAFLD. CO was also capable of restoring mitochondrial ultrastructure and function as well as balance superoxide dismutase and catalase levels. Our findings indicate that CO treatment has positive effects on NAFLD improving mitochondrial dysfunction, steatosis, acute inflammation, and oxidative stress.

## 1. Introduction

Nonalcoholic fatty liver disease (NAFLD) is the accumulation of fat in the liver in patients who do not consume excessive alcohol [1]. The term NAFLD encompasses a wide spectrum of conditions, from simple accumulation of fat (“fatty liver” or steatosis) to steatohepatitis, fibrosis, and cirrhosis with its clinical consequences [2]. NAFLD affects about 30% of the general population in western society and is recognized as the most common cause of liver dysfunction worldwide [3, 4].

NAFLD is asymptomatic in most affected patients and characterized as the presence of more than 5% of lipid accumulation in the hepatocytes, excluding other liver disease etiologies (virus, autoimmune, alcohol, drugs, and genetics). NAFLD can progress to an advanced form, nonalcoholic steatohepatitis (NASH), which is defined by histological findings of hepatic steatosis, with hepatocyte damage and or inflammation [5–7]. Although uncommon, both NAFLD and NASH can further develop into liver fibrosis, cirrhosis, and eventually hepatocellular carcinoma [8, 9].

In addition to genetic risk factors, the prevalence of NAFLD is aggravated by changes in life style including physical inactivity as well as consumption of high fat and high fructose corn syrup diet [10]. Recently, NAFLD has been considered as a hepatic manifestation of metabolic syndrome (MS) which is defined by the presence of central obesity, insulin resistance, hyperlipidemia, hyperglycemia, and hypertension [3, 11–13]. NAFLD induces insulin resistance and exacerbates systemic chronic inflammation and oxidative stress, which leads to organ dysfunction in extrahepatic tissues. There is now growing evidence that NAFLD is actually a multisystem disease, affecting several extrahepatic organs and regulatory pathways [14, 15].

Evidence suggests that NAFLD might be a mitochondrial disease. Mitochondrial dysfunction seems to play a role in the pathogenesis of NAFLD since it affects hepatic lipid homeostasis and promotes reactive oxygen species (ROS) production and lipid peroxidation, cytokine release, and cell death [16–18]. Mice with mitochondrial dysfunction develop NAFLD and hepatic insulin resistance independent of obesity or high-fat diet (HFD) [19, 20].

Although there is no specific effective treatment available for NAFLD, dietary therapy already showed beneficial results [21]. Administration of polyunsaturated fatty acids (PUFA) improves plasma lipid profile and may be useful in the treatment of NAFLD [22, 23]. A recent systematic review and meta-analysis about the efficacy of PUFA in NAFLD derived from clinical trials confirmed an improvement in patients with this condition. The clinical improvement was based on hepatic steatosis and no other clinical or histological aspects were evaluated [24].

The oil derived from capybara* (Hydrochoerus hydrochaeris)* (CO), the largest rodent in the world, is a rich source of PUFA and contains 17.9% of alpha-linoleic and 19.6% linoleic fat acids (FAs), which correspond to essential omega-3 (n-3) and omega-6 (n-6) FAs, respectively. In a previous study, CO reduced cholesterol blood levels in rats fed with cholesterol-enriched diets [25]. The aim of this study was to determine the effect of CO on hepatic mitochondrial dysfunction, steatosis, and inflammation in a murine model of NAFLD.

## 2. Materials and Methods

### 2.1. Animals and Capybara Oil

This study was performed according to protocols (CEUA/015/2013) approved by the local Ethics Committee for Use and Care of Experimental Animals of the State University of Rio de Janeiro. All animals were maintained in 21 ± 2°C, 60%  ± 10% of humidity, and 12 : 1 dark-light cycle. Capybara fat was donated by a private slaughter farm in Brazil that breed capybara in captivity as authorized by the Brazilian Institute of Environment and Renewable Natural Resources. The CO composition was previously described [25]. Oil was extracted using hydrothermal processing of the fat in a water bath [26].

### 2.2. NAFLD Induction and Capybara Oil Treatment

Thirty C57BL/6 male mice (8 weeks old) were divided into three groups (*n* = 10 per group): control group (CG), NAFLD group (NAFLD), and NAFLD treated with CO group (NAFLD + CO). CG animals were fed a control diet (70% carbohydrates, 10% fat, and 20% proteins), and NAFLD animals were fed a HFD (29% carbohydrates, 55% fat, and 16% proteins) during 16 weeks in order to induce NAFLD [27]. The NAFLD + CO group received CO by oral gavage (1.5 g/kg/daily) during the last 4 weeks of the diet protocol while CG and NAFLD groups received water by gavage. One day after the last day of treatment, the animals were euthanized.

### 2.3. Sample Collection

After the last day of treatment, mice were fasted for 6 hours and anesthetized (xylazine 2 mg/Kg and ketamine 5 mg/Kg). Blood samples were collected by cardiac puncture and transferred to heparinized tubes. Samples were then centrifuged (1500 g/10 min) to obtain plasma which were stored at −80°C until biochemical analyses. Liver samples were also collected and handled according to the specific techniques. Some samples were immediately used for high resolution respirometry while others were either frozen or fixed in different solutions for further analyses.

### 2.4. Serum Liver Enzymes

Plasma liver enzymes aspartate aminotransferase (AST) and alanine aminotransferase (ALT) were measured by a colorimetric enzymatic assay according to manufacturer's recommendations (Bioclin, Brazil).

### 2.5. Steatosis Quantification

Liver fragments fixed in paraformaldehyde (4% w/v) were embedded in paraffin, sectioned at a nominal thickness of five *μ*m, and stained with hematoxylin and eosin (HE). Ten digital images per animal were analyzed in a random and blinded manner and studied to assess steatosis as previously described [28]. Briefly, we used a 36-point test-system (PT) produced by the STEPanizer web-based software (http://www.stepanizer.com). The volume density (Vv) of liver steatosis was estimated by point-counting the fat droplets in hepatocytes: Vv [steatosis] = PP [steatosis]/PT (2) where PP is the number of points that defined steatosis [28, 29]. This method was proved to be more consistent in assessing the hepatic steatosis than the use of scores [28].

### 2.6. Immunohistochemistry

Liver sections had antigen retrieval performed by citrate buffer at pH 6.0 incubation for 30 min at 60°C. Endogenous peroxidase activity was blocked using 0.3% hydrogen peroxide (H_2_O_2_) and nonspecific binding of the polyclonal antibodies was blocked by incubation 5% (w/v) bovine serum albumin (BSA) diluted in phosphate buffered saline (PBS). Subsequently, sections were incubated with antibodies, and these reactions were amplified using a biotin-streptavidin system (Dako, USA). Immunoreactive products were visualized using diaminobenzidine (DAB) reagent (Dako) and counterstained with hematoxylin. We used antitumor necrosis factor alpha (TNF-*α*), and antitransforming growth factor beta (TGF-*β*) (dilution 1 : 100, Dako) antibodies. The estimated density per area (*Q*_*A*_) of TNF-*α* and TGF-*β* positive cells was defined as cell numerical profile within a known test area (expressed as cells/*μ*m^2^) of 3305 *μ*m^2^ [26] using Image Pro Plus software. All analysis was held in blind test.

### 2.7. Ultrastructural Analyses

Liver samples were fixed in 2.5% glutaraldehyde (Sigma-Aldrich, Germany) in 0.1 mol/L cacodylate buffer, post-fixed in 1% osmium tetroxide (Sigma-Aldrich), and embedded in Epon (Embed-812, USA). Ultrathin sections (60 to 70 nm) were obtained from selected areas using an ultramicrotome (Leica, Austria), contrasted with uranyl acetate and lead citrate, and examined with a transmission electron microscope (TEM) (Jeol 1001, Japan) at 80 kV.

### 2.8. Oxidative Stress Analyses

Frozen liver homogenates were used for biochemical assays, as performed by Kennedy-Feitosa et al., 2016 [30]. Superoxide dismutase (SOD) activity was assayed by monitoring adrenaline (Sigma-Aldrich, USA) inhibition autooxidation. Catalase (CAT) activity was measured by a decrease of H_2_O_2_ (Sigma-Aldrich, USA) rate, and concentrations were monitored. As an index of oxidative damage induced by lipid peroxidation, we used thiobarbituric acid reactive substances (TBARS) (EMD Millipore, USA) method to analyze malondialdehyde (MDA) products during an acid-heating reaction. TBARS levels were expressed as MDA equivalents.

### 2.9. Mitochondrial Respirometry

We used high-resolution respirometry protocol according to Cortez et al., 2012 [31]. Briefly, liver fragments were mechanically macerated by using sharpened forceps and transferred into vessels with cooled Mitochondrial Respiration Medium, MIR05. Respiratory rates of liver were measured with Oroboros 2k-Oxygraph (Oroboros Instruments, Austria) in 2 ml of MIR05 at 37°C with continuous stirring. Matlab software (Oroboros Instruments) was used for data acquisition and analysis. Studies were performed with two independent sets of substrates, the carbohydrate (CHO) and FA protocol. Respiratory parameters were defined as follows: maximally adenosine diphosphate- (ADP-) (5 mM) stimulated respiration rates (State 3) and respiration rates in ADP phosphorylation absence and measured in presence of 1 *μ*g/ml oligomycin (State 4). From respiratory fluxes obtained during substrate titration protocol, respiratory control ratio (RCR) was calculated for State 3/State 4.

### 2.10. Statistical Analyses

A statistical software (GraphPad Prism 6) was used for statistical analyses and graphics generation. Data is presented as means ± standard error of the mean (SEM). Analysis of variance (one-way ANOVA) was performed followed by Bonferroni test. A *p* value less than 0.05 was considered statistically significant.

## 3. Results

### 3.1. Serum Liver Enzymes

The levels of ALT (Table 1) were significantly increased (*p* < *p* = 0.026) in the NAFLD group (25.0 ± 6.4 mg/dL) compared to the CG group (6.8 ± 0.9 mg/dL). Four weeks of treatment with CO reduced ALT levels in 46.4% (13.4 ± 3.8). No differences were found in the levels of AST (Table 1) between groups (CG = 55.0 ± 4.9; NAFLD = 59.0 ± 5.9; NAFLD + CO = 57.8 ± 4.5).

### 3.2. Steatosis Quantification

**Figure 1:**
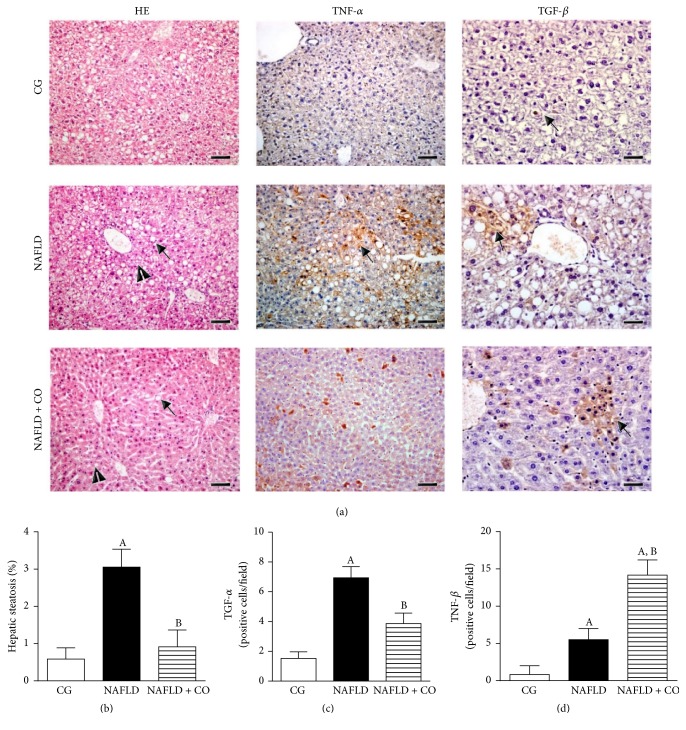
*Morphological changes in the liver of C57BL/6 mice treated with capybara oil*. (a, b, c, and d) (a) Panel with a photomicrograph of liver showing hepatic steatosis (HE), tumor necrosis factor-alpha (TNF-*α*) and transforming growth factor-beta (TGF-*β*) expression. The black arrows indicate the macrovesicles and arrows head indicate the microvesicles (HE); the black arrows indicate immunostaining of TNF-*α* and immunostaining of TGF-*β*, respectively. Bar indicates 400 *μ*m to HE and TNF-*α* images and 200 *μ*m to TGF-*β* images. (b) Evaluation of hepatic steatosis by morphometry. (c) Quantification of TNF-*α* by Immunohistochemistry. (d) Quantification of TGF-*β* by Immunohistochemistry. Data are expressed as the mean ± SEM. Statistical analyses were performed using a one-way ANOVA analysis of variance followed by Bonferroni test. (b) ^A^*p* = 0.0006 compared to the CG group and ^B^*p* = 0.0021 compared to NAFLD group. (c) ^A^*p* < 0.0001 compared to the CG group and ^B^*p* = 0.0042 compared to NAFLD group. (d) ^A^*p* = 0.0002 compared to the CG group (in the NAFLD bar); ^A^*p* < 0.0001 compared to the CG group (in NAFLD + CO bar) and ^B^*p* < 0.0001 compared to NAFLD group.

Animals from CG showed a low number of fat droplets (Figure 1(a)) and a small steatosis percentage (CG = 2.94 ± 1.35%) (Figure 1(b)). Animals fed a high fat diet without treatment showed severe macro- and microlipid droplets in hepatocytes (Figure 1(a)) and significantly increasing steatosis density (NAFLD = 18.89 ± 2.32%) (Figure 1(b)). Treatment with CO decreased the severity of lipid droplets in hepatocytes (Figure 1(a)) and consequently the percentage of fat accumulation (NAFLD + CO = 4.97 ± 1.92%) (Figure 1(b)).

### 3.3. Immunohistochemistry

Immunohistochemistry staining was performed in liver tissue to analyze the effect of CO on the inflammatory response induced by NAFLD. TNF-*α* expression significantly increased in NAFLD group (6.93 ± 0.75 positive cells/fields) compared to CG (1.53 ± 0.44 positive cells/fields) (Figure 1(a)). Likewise, the expression of TGF-*β* increased significantly in NAFLD group (5.50 ± 0.62 positive cells/fields) compared to CG (0.83 ± 0.47 positive cells/fields) (Figure 1(a)). Treatment with CO significantly decreased the TNF-*α* expression (NAFLD + CO = 3.87 ± 0.70 positive cells/fields) (Figure 1(c)) but greatly increased TGF-*β* expression (NAFLD + CO = 14.17 ± 0.83 positive cells/fields) (Figure 1(d)).

### 3.4. Ultrastructural Liver Analyses

**Figure 2:**
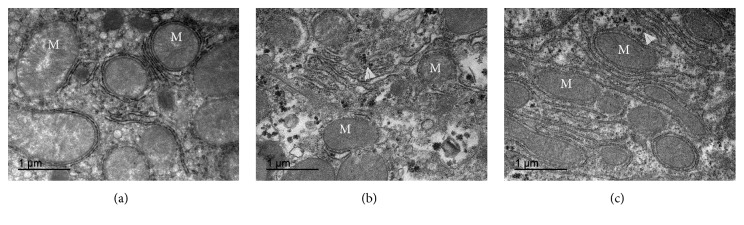
*Mitochondrial morphological evaluation in the liver of C57BL/6 mice treated with capybara oil*. (a, b, and c) Panel with ultraphotomicrograph of hepatocytes showing the mitochondrial morphology of CG, NAFLD, and NAFLD + CO, respectively; *M* = mitochondria. Whitehead arrows indicate glycogen. Bars indicate 1 *μ*m.

The electron microscopy analyses of CG showed preserved organelles and numerous well developed and organized mitochondria with an intact membrane and matrix (Figure 2(a)). The NAFLD group presented a decrease in mitochondrial matrix electron density and loss of membrane integrity. This membrane disruption leads to consequent mitochondrial matrix extravasation in the cytoplasm. Additionally, NAFLD group showed a remarkable disorganization in the cytoarchitecture and higher accumulation of glycogen in the cytoplasm (Figure 2(b)). On the other hand, hepatocytes of animals treated with CO exhibited numerous mitochondria and recovery of the mitochondrial membrane integrity and matrix electron density. Hepatic cells from NAFLD + CO group remained with organized ultrastructural arrangement compared to NAFLD group and showed less glycogen accumulation (Figure 2(c)).

### 3.5. Oxidative Stress Analysis

**Figure 3:**
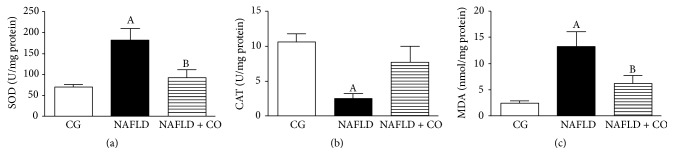
*Oxidative stress evaluation in the liver of C57BL/6 mice treated with capybara oil*. (a, b, and c) (a–c) represents antioxidants enzymes and lipid peroxidation products in serum, (a) superoxide dismutase (SOD) activity, (b) catalase (CAT) activity, and (c) malondialdehyde (MDA) levels. Data are expressed as mean ± SEM. Statistical analyses were performed using a one-way ANOVA analysis of variance followed by Bonferroni test. (a) ^A^*p* = 0.0084 compared to CG group; ^B^*p* = 0.0364 compared to NAFLD group. (b) ^A^*p* = 0.0035 compared to CG group. (c) ^A^*p* = 0.0006 compared to CG group; ^B^*p* = 0.0264 compared to NAFLD group.

NAFLD group showed significantly increased SOD antioxidant enzyme activity (180.9 ± 27.46 U/mg protein) compared to the CG group (70.09 ± 5.35 U/mg protein). Treatment with CO significantly decreased SOD activity (NAFLD + CO = 91.56 ± 19.42 U/mg protein) compared with NAFLD group (Figure 3(a)). NAFLD group showed depressed CAT antioxidant enzymes activity (2.53 ± 0.63 U/mg protein) compared to CG group (10.6 ± 1.14 U/mg protein). No significant difference was found in CAT activity after CO treatment (NAFLD + CO = 7.66 ± 2.32 U/mg protein) compared to NAFLD group (Figure 3(b)). NAFLD group showed significantly increased in the MDA levels (13.16 ± 2.83 nmol/mg protein) compared to the CG group (2.41 ± 0.48 nmol/mg protein). Treatment with CO significantly decreased MDA levels (NAFLD + CO = 6.22 ± 1.53 nmol/mg protein) compared with NAFLD group (Figure 3(c)).

### 3.6. Mitochondrial Respirometry

**Figure 4:**
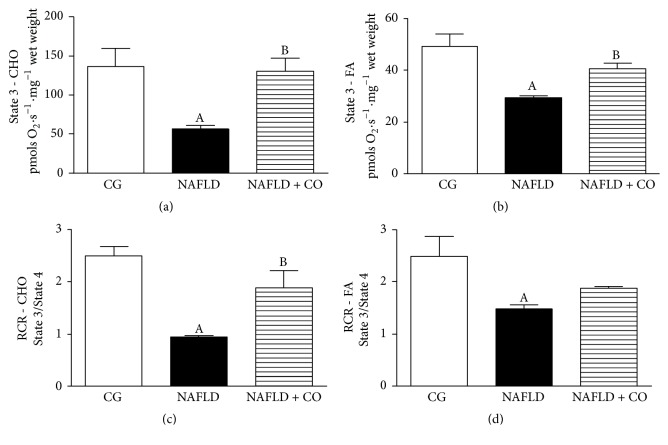
*Mitochondrial functional evaluation in the liver of C57BL/6 mice treated with capybara oil*. (a, b, c, and d) (a) represents high-resolution respirometry in liver cells. (b) Maximal ADP-stimulated respiratory rates (State 3) with substrates related to carbohydrate oxidation, (c) maximal ADP-stimulated respiratory rates (State 3) with substrates related to fatty acid oxidation, (d) respiratory control ratio (RCR), and calculated as State 3/State 4 ratios for CHO and FAs oxidation, respectively. Data are expressed as mean ± SEM. Statistical analyses were performed using a one-way ANOVA analysis of variance followed by Bonferroni test. (a) ^A^*p* = 0.0212 compared to CG group; ^B^*p* = 0.0286 compared to NAFLD group. (b) ^A^*p* = 0.0019 compared to CG group; ^B^*p* = 0.0472 compared to NAFLD group. (c) ^A^*p* = 0.0005 compared to CG group; ^B^*p* = 0.0187 compared to NAFLD group. (d) ^A^*p* = 0.0225 compared to CG group.

To evaluate mitochondrial function, respirometry experiments were performed. NAFLD animals without treatment showed liver mitochondrial impairment, with a reduction in maximum ADP-stimulate respiratory rates (State 3) in oxidation protocols, CHO, and FAs reduction, compared with CG. CO treatment (NAFLD + CO) promoted a significant increase in State 3 respiration in CHO and FAs, respectively (Figures 4(a) and 4(b)). Furthermore, RCR was significantly decreased in CHO and FAs oxidation in NAFLD group compared to CG. After CO treatment an increase in RCR levels was observed similar to CHO oxidation in CG (Figure 4(c)). The CO effect was not verified in relation to FAs oxidation (Figure 4(d)).

## 4. Discussion

The present study showed that CO, an alternative PUFA source, was capable of reversing liver damage associated with NAFLD in obese mice. The oil was able to reduce plasmatic levels of ALT enzyme, decrease hepatic steatosis, and improve tissue morphology and inflammation. Moreover, CO improved abnormalities in mitochondrial ultrastructure, cellular respiratory efficiency, and imbalance of antioxidant enzymes.

Prolonged HFD in C57Bl/6 mice is an established model of diet-induced obesity [32]. Previous studies have demonstrated that HFD also leads to NAFLD [33, 34] and in humans can sometimes evolve to NASH [35]. As expected, animals submitted to the present HFD successfully developed features of NAFLD including liver steatosis, increased plasma liver enzymes, and inflammation compared with control animals. Although this model of NAFLD mimics both the histopathology and pathogenesis of human NAFLD [36] animals do not develop NASH or fibrosis. Further investigation is necessary to determine if CO has any beneficial effects on progressive forms of NAFLD that may lead to fibrosis, cirrhosis, and ultimately hepatocellular carcinoma.

Aminotransferase levels have been used as a marker of NAFLD [37]. NAFLD is the commonest cause of elevated ALT and presumed liver injury [38, 39]. ALT and/or AST levels seem to be predictive of the presence of NAFLD when other chronic liver diseases are excluded and features of MS like obesity are present [38]. There are two soluble ALT isoenzymes in mammals, cytosolic and mitochondrial, playing a role in amino acid metabolism and gluconeogenesis [40]. CO treatment successfully reduced plasmatic levels of ALT suggesting a recovery of enzyme leakage from damaged hepatocytes.

Fat accumulation in the liver of patients with NAFLD is mainly in the form of triglycerides [41, 42]. Triglyceride accumulation is not hepatotoxic per se and could represent a defensive mechanism to balance free fat acid (FFA) excess [43, 44]. Previous evidences suggest that liver steatosis is an epiphenomenon, which happens simultaneously with toxic metabolites generation, lipotoxicity, and liver injury. Our data shows that CO treatment reduced liver steatosis in 73.69%, which agrees with previous studies using PUFA in the treatment of NAFLD [22–24].

NAFLD is associated with the production and release of proinflammatory cytokines, both systemically and locally in the liver, such as TNF-*α* [45]. The persistence of this cytokine could lead to chronic inflammation with fibrosis development, as characterized in NASH condition [46]. Serum and hepatic levels of TNF-*α* are increased in patients with NASH and correlate with histological severity of liver damage [47]. We found hepatic levels of TNF-*α* to be increased in our model and CO significantly reduced hepatic TNF-*α* levels. The absence of NASH and fibrosis in our model is a limitation of this study. Nevertheless, this cytokine is known to contribute to mitochondrial dysfunction, inducing mitochondrial swelling, rarefaction of the matrix, and membrane burst interfering in mitochondrial respiratory complexes [48]. We also investigated the hepatic levels of TGF-*β*, a pleiotropic cytokine that plays a dual role in immune functions [49]. Biological actions of TGF-*β* include regulation of cell proliferation, control of extracellular matrix protein production and degradation, cellular differentiation modulation, and either stimulatory or inhibitory effects on the same cell, depending on the cellular environment [50]. It is well stablished that n-3 PUFA is able to interact with peroxisome proliferator-activated receptor *α* (PPAR*α*) and inhibit factor nuclear kappa B (NF-*κ*B) [51], resulting in reduction of TNF-*α* and interleukin (IL-6) expression in hepatocytes [52]. In adaptive immune response, the differentiation of (cluster of differentiation) CD4^+^ T cells subtypes in Th17 proinflammatory T cells is dependent of association between IL-6 and TGF-*β*, while the anti-inflammatory Treg cells only need high levels of TGF-*β* stimulation to differentiate [53]. This mechanism could be related to our data, suggesting that CO treatment, rich in n-3 PUFA, helped to decrease inflammation either through inhibition of NF-*κ*B (indirectly indicated by the decrease in TNF-*α* production) or by increasing TGF-*β* levels in the liver (and probably the anti-inflammatory response stimulating Treg cells differentiation). However, more analyses are necessary to confirm this hypothesis.

Structural and functional alterations in mitochondria have showed to contribution in the pathogenesis of NAFLD. Structural alterations encompass depletion of mitochondrial deoxyribonucleic acid (DNA) and morphological and ultrastructural changes, while functional alterations include the respiratory chain and mitochondrial *β*-oxidation [52]. The mitochondrial respiratory efficiency loss seems to occur due to a possible uncoupling effect exerted by the excess of FAs present in the HFD [54, 55]. We found several structural and functional derangements in our animals with NAFLD that were efficiently treated by CO. Whether mitochondrial dysfunction is a key pathogenic event in NAFLD or the consequence of an altered lipid metabolism remains unclear.

Oxidative stress results from an imbalance between ROS, prooxidant, and antioxidant chemical species that leads to cellular oxidative damage. The FAs oxidation is an important source of ROS in fatty livers [56] and oxidative stress exacerbation in NASH promotes hepatocellular damage by inducing severe alterations in biomolecules, with functions loss and cell viability [57]. The mitochondrial respiration dysfunction, observed in our study, can directly lead to ROS production. If electron flow is interrupted; respiratory intermediates transfer electrons to molecular oxygen producing superoxide anions and H_2_O_2_. With impaired mitochondrial oxidative capacity, cytosolic FAs accumulate [48]. The decrease of antioxidant CAT enzyme activities, found in the NAFLD group, seems to occur because of rapid consumption and exhaustion of these enzymes storage, a consequence of their role in avoiding free radicals generated in NAFLD. In our study, SOD enzyme activity was higher in NAFLD group in agreement with previous report using high fat diet [58]. High concentrations of antioxidant enzymes represent stress oxidation that the body tries to eliminate [59]. NAFLD increased liver oxidative stress as evidenced by the increase liver's oxidative damage (such as the increase in MDA level) and the inhibition of liver's antioxidant enzymes (CAT) [58]. Treatment with CO was able to significantly reverse MDA levels, consequently lipid peroxidation, and oxidative stress.

Recently, there was an increasing interest in the use of the PUFAs as a nutritional supplementation, since many studies have been proving protective effects in patients with different diseases including the ones related to obesity [60, 61]. Although some authors have found that PUFA treatment seems to be not effective in NASH [62, 63], its effects in early stages of the disease is undeniable reversing the hallmark features observed in human NAFLD patients. Whether or not the prevention of NALFD progression to NASH is relevant when treating obese patients is something that requires further investigation.

In most of the studies, PUFA sources are fish or plants where the extraction procedures are complex and result in low yields of the product [59, 60, 64, 65]. We decided to investigate the CO, due to its high levels of PUFA and potential for becoming an alternative source of these FAs [25]. This oil has potential to be used for consumption in the food industry, rather than being discarded, as capybara meat is already marketed for consumption. Additional to the previous study showing beneficial effects on dyslipidemia [25], our group has also described that CO acts beneficially in cutaneous wound healing [26].

## 5. Conclusion

We conclude that CO treatment has positive effects on the benign form of obesity-induced NAFLD improving mitochondrial dysfunction, steatosis, acute inflammation, and oxidative stress in mice.

## Figures and Tables

**Table 1:** Plasma liver enzymes of C57BL/6 mice treated with capybara oil.

Parameters	Groups			
	CG	NAFLD	NAFLD + CO	
ALT (mg/dl)	6,8 ± 0,9	25,0 ± 6,4^a^	13,4 ± 3,8^b^	
AST (mg/dl)	55,0 ± 4,9	59,0 ± 5,9	57,8 ± 4,5	

Alanine aminotransferase (ALT); aspartate aminotransferase (AST); control group (CG); NAFLD group (NAFLD); NAFLD treated with CO group (NAFLD + CO). Data were expressed as mean ± SEM. Statistical analyses were performed using a one-way ANOVA analysis of variance followed by Bonferroni test. a represents *p* = 0.026 compared to CG group and b represents *p* = 0.046 compared to NAFLD group.

## Data Availability

The data used to support the findings of this study will be available upon request. Please contact Dr. Jorge José de Carvalho, Head of the Laboratory of Ultrastructure and Tissue Biology at carvalho@uerj.edu.br.
